# Chemosensory signalling pathways involved in sensing of amino acids by the ghrelin cell

**DOI:** 10.1038/srep15725

**Published:** 2015-10-29

**Authors:** L. Vancleef, T. Van Den Broeck, T. Thijs, S. Steensels, L. Briand, J. Tack, I. Depoortere

**Affiliations:** 1 Gut Peptide Research Lab, Translational Research Center for Gastrointestinal Disorders, Department of Clinical & Experimental Medicine, University of Leuven, Leuven, 3000, Belgium; 2 INRA UMR1324, CNRS UMR6265, Université de Bourgogne, Centre des Sciences du Goût et de l’Alimentation, F-21000, Dijon, France

## Abstract

Taste receptors on enteroendocrine cells sense nutrients and transmit signals that control gut hormone release. This study aimed to investigate the amino acid (AA) sensing mechanisms of the ghrelin cell in a gastric ghrelinoma cell line, tissue segments and mice. Peptone and specific classes of amino acids stimulate ghrelin secretion in the ghrelinoma cell line. Sensing of L-Phe occurs via the CaSR, monosodium glutamate via the TAS1R1-TAS1R3 while L-Ala and peptone act via 2 different amino acid taste receptors: CaSR & TAS1R1-TAS1R3 and CaSR & GPRC6A, respectively. The stimulatory effect of peptone on ghrelin release was mimicked *ex vivo* in gastric but not in jejunal tissue segments, where peptone inhibited ghrelin release. The latter effect could not be blocked by receptor antagonists for CCK, GLP-1 or somatostatin. *In vivo*, plasma ghrelin levels were reduced both upon intragastric (peptone or L-Phe) or intravenous (L-Phe) administration, indicating that AA- sensing is not polarized and is due to inhibition of ghrelin release from the stomach or duodenum respectively. In conclusion, functional AA taste receptors regulate AA-induced ghrelin release *in vitro*. The effects differ between stomach and jejunum but these local nutrient sensing mechanisms are overruled *in vivo* by indirect mechanisms inhibiting ghrelin release.

Obesity is one of the major healthcare problems of our western society reaching epidemic proportions. To date, gastric bypass surgery is the only intervention that consistently induces sustained weight loss and remission of concomitant diseases^1^,^2^. A less drastic tool to induce weight loss is focussing on the macronutrient composition of a diet.

A high-protein diet has beneficial effects on both weight loss and weight maintenance compared to an isoenergetic intake of fat and carbohydrates^3^. A large European study showed that a diet with a modest increase in protein content and a modest reduction in glycaemic index led to an improvement in maintenance of weight loss^4^. It is already known that dietary protein intake results in the release of satiety hormones like peptide YY (PYY), glucagon-like peptide 1 (GLP-1) and cholecystokinin (CCK)^5^. Studies in PYY null mice showed an important role for PYY in protein-mediated satiation and body weight regulation^6^. In addition, several studies reported a decrease in plasma ghrelin levels following ingestion of a high-protein diet^7^,^8^.

Ghrelin is a 28-amino acid peptide released from the X/A-like cells of the gastric oxyntic mucosa in response to hunger and starvation^9^. A posttranslational modification by the enzyme ghrelin-O-acyltransferase (GOAT), placing an octanoyl group on the third amino acid serine (Ser^3^), is required for ghrelin’s biological activity^10^,^11^. Although initially discovered as a hormone that stimulates growth hormone secretion, ghrelin is now considered as a multifunctional hormone that stimulates food intake, prevents fat utilization, increases body weight, inhibits glucose-induced insulin release and stimulates gastrointestinal motility^9^,^12^,^13^,^14^.

The chemosensory signalling pathways involved in sensing of peptides and amino acids by the ghrelin cell are unknown. Specific amino acid taste receptors on enteroendocrine cells, similar to those in the lingual system, and transporters on enterocytes in the gut are candidates for sensing and uptake of protein breakdown products^15^,^16^. Up to now, three important amino acid taste receptors are cloned and characterized, each with a specific preference for a particular class of amino acids. The calcium sensing receptor (CaSR), expressed in gastrin- (G-cells), cholecystokinin- (CCK) (I-cells) and somatostatin-secreting cells (D-cells), mainly senses aromatic amino acids and calcium (Ca^2+^)^17^,^18^,^19^. The G-protein coupled receptor family C group 6 member A (GPRC6A), a receptor that predominantly senses basic amino acids and Ca^2+^, acts in concert with the CaSR and is expressed in gastric antral D- and G-cells^18^,^20^. The heterodimer taste receptor type 1 member 1 - taste receptor type 1 member 3 (TAS1R1-TAS1R3), also known as the umami taste receptor, is mainly activated by umami stimuli, glutamic and aspartic acids, in humans but in rodents it broadly detects most of the 20 L-amino acids^21^,^22^. One of the subtypes, TAS1R3, which is also involved in sensing of carbohydrates, is expressed in L-cells and X/A-like cells^23^,^24^. Besides TAS1R1-TAS1R3, also metabotropic glutamate receptors, present in the stomach and small intestine, can sense umami taste^25^. Peptone, an enzymatic digest from animal milk or protein mimicking dietary protein digests in the lumen, is likely sensed by the lysophosphatidic acid receptor 5 (LPAR5; also known as GPR92 and GPR93)^26^. Furthermore, free amino acids can be absorbed via various amino acid transporters with different group specificities whereas di- and tripeptides are taken up by the peptide transporter PepT1^27^,^28^. Studies in Pept1^−/−^ mice showed that PepT1 participates in the control of food intake in mice fed a high-protein diet^28^. Collectively, these data suggest that sensing of amino acids and protein hydrolysates by endocrine cells in the gut is finely tuned by different receptors and transporters that may play an important role in protein-induced satiety.

Recent studies showed that the ghrelin cell expresses TAS1R3, involved in sensing of sweet and umami, but also the free fatty acid receptor GPR120 and the gustatory G-proteins, α-gustducin and α-transducin, coupled to several taste receptors^23^,^29^,^30^. This study aimed to examine the role of specific amino acid taste receptors in the effect of peptone, a casein hydrolysate rich in peptides and amino acids, and individual amino acids on ghrelin release in the ghrelinoma cell line. Since ghrelin cells exist as closed-type cells in the stomach, not reaching the epithelial surface, or opened-type cells in the small intestine, making contact to the lumen, we investigated whether the effect of peptone on ghrelin release differed in *ex vivo* segments from corpus and jejunum^31^. Finally, we examined whether amino acids are sensed via the lumen or bloodstream by comparing the effect of intragastric versus intravenous administration of peptone or L-Phe on ghrelin release in mice.

## Results

### *In vitro* studies in the ghrelinoma cell line

#### MGN3-1 cells release octanoyl ghrelin in response to a casein hydrolysate and L-amino acids

**Figure 1:**
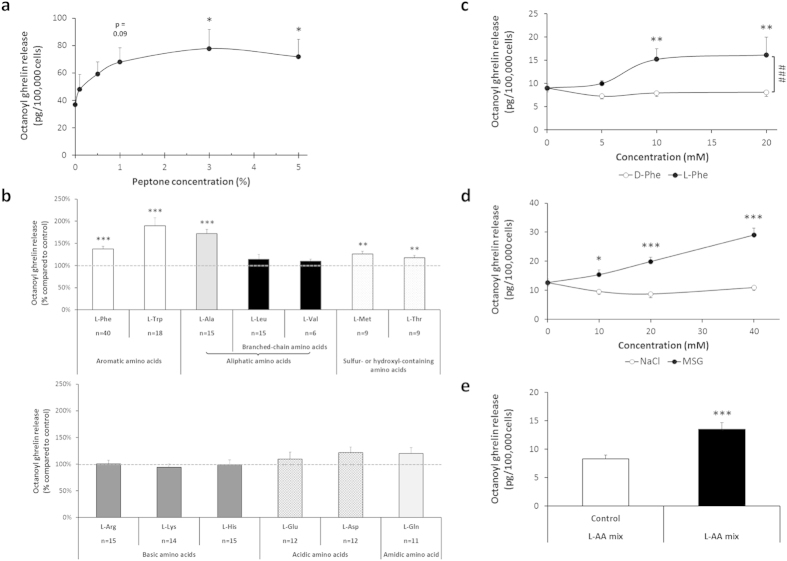
Effect of peptone and amino acids on octanoyl ghrelin release in MGN3-1 cells. (**a**) Concentration-dependent effect of a 3 h stimulation with peptone on octanoyl ghrelin release (n = 8). (**b**) Effect of amino acids from 7 different classes (10 mM) on octanoyl ghrelin release compared to the control stimulation (HEPES buffer) in each condition. (**c**) Stereoselective effect of L-Phe on octanoyl ghrelin release (n = 3–11). (**d**) Concentration-dependent effect of MSG on octanoyl ghrelin release compared to NaCl as control (n = 5–6). (**e**) Effect of an amino acid mixture (L-AA mix), mimicking the fasting levels of amino acids in human plasma, on octanoyl ghrelin release (n = 12) compared to HEPES buffer as control. *P < 0.05 vs. control; **P < 0.01 vs. control; ***P < 0.001 vs. control; ^###^*P* < 0.001 vs. D-Phe.

The effect of peptone and of different classes of amino acids on octanoyl ghrelin release was investigated in a ghrelinoma cell line, MGN3-1. Peptone stimulated octanoyl ghrelin release in a concentration-dependent manner with maximal effects at 3% (P = 0.01) and 5% peptone (P = 0.04) (Fig. 1a). The aromatic amino acids L-Phe and L-Trp stimulated octanoyl ghrelin release with 37 ± 6% (P = 0.000001) and 90 ± 18% (P = 0.00005) respectively (Fig. 1b). L-Phe increased ghrelin levels between 5 and 10 mM with no further increases up to 20 mM. Since natural proteins are exclusively built from L-amino acids and amino acid taste receptors are only selective for L-amino acids^22^,^32^, the effect of D-Phe was tested to determine the specificity of the observed effect. D-Phe was without effect up to 20 mM, indicating that the effect of L-Phe was indeed stereoselective (Fig. 1c). The aliphatic amino acid L-Ala 10 mM (P = 0.000007) and the sulphur- or hydroxyl-containing amino acids L-Met 10 mM (P = 0.002) and L-Thr 10 mM (P = 0.0098) significantly increased octanoyl ghrelin release. None of the other classes of amino acids (branched-chain, basic, acidic and amidic amino acids) were effective at 10 mM (Fig. 1b). Monosodium glutamate (MSG), known for its specific umami taste, increased octanoyl ghrelin release, compared to NaCl as control, in a concentration-dependent manner (P = 0.00001) without reaching a plateau at 40 mM (Fig. 1d). Application of a mixture of L-amino acids, mimicking the fasting levels of amino acids in human plasma, increased (P = 0.0009) octanoyl ghrelin release with 63 ± 14% (Fig. 1e).

### The effect of individual amino acids on octanoyl ghrelin release in the ghrelinoma cell line is mediated by different amino acid taste receptors

**Figure 2:**
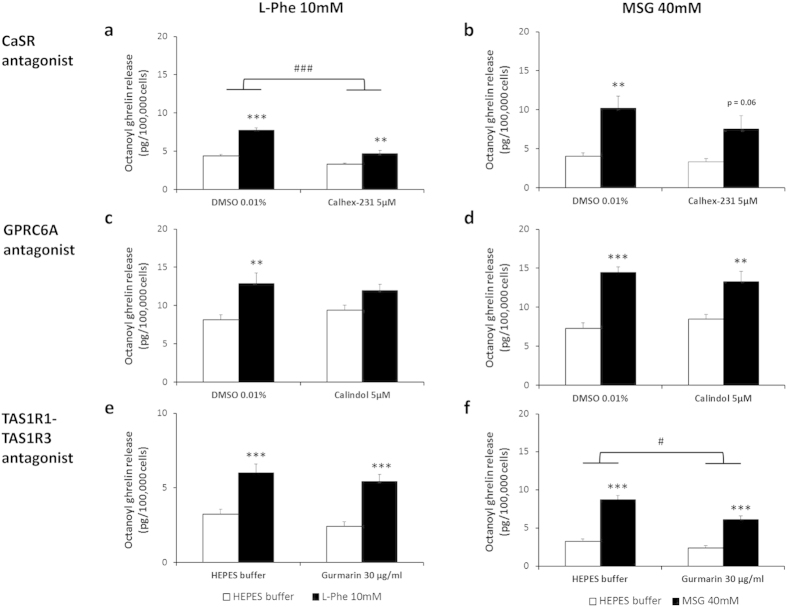
Effect of amino acid taste receptor blockers on L-Phe- and MSG-induced octanoyl ghrelin release in MGN3-1 cells. Effect of preincubation (30 min) of MGN3-1 cells with a (**a**,**b**) CaSR antagonist (calhex-213 5 μM) (n = 6–15), (**c**,**d**) GPRC6A antagonist (calindol 5 μM) (n = 9–12) or (**e**,**f**) TAS1R1-TAS1R3 antagonist (gurmarin 30 μg/ml) (n = 9) or their respective vehicle on the effect of (**a**,**c**,**e**) L-Phe 10 mM or (**b**,**d**,**f**) MSG 40 mM compared to HEPES buffer on octanoyl ghrelin release in MGN3-1 cells. **P < 0.01, ***P < 0.001 vs. vehicle or vehicle with antagonist; ^#^*P* < 0.05, ^###^*P* < 0.001 in the absence vs. presence of antagonist.

**Figure 3:**
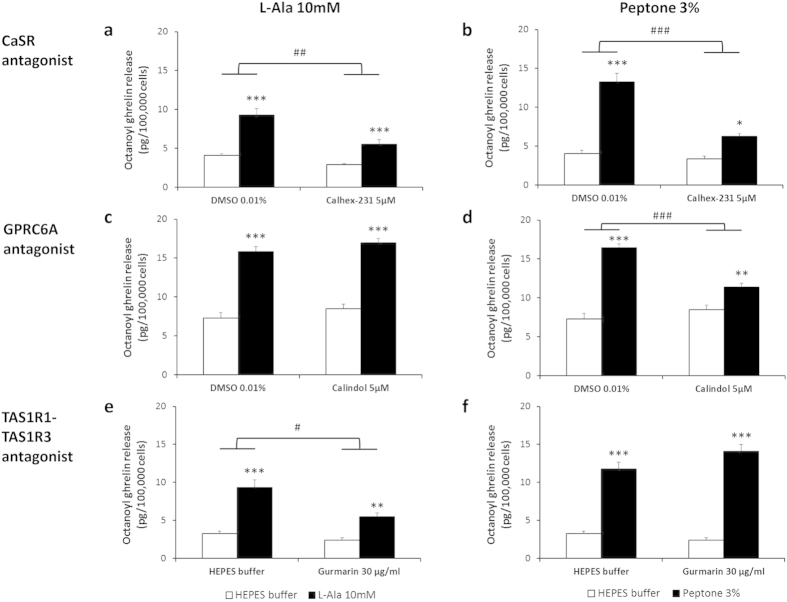
Effect of amino acid taste receptor blockers on L-Ala- and peptone-induced octanoyl ghrelin release. Effect of preincubation (30 min) of MGN3-1 cells with a (**a**,**b**) CaSR antagonist (calhex-213 5 μM) (n = 6–9), (**c**,**d**) GPRC6A antagonist (calindol 5 μM) (n = 9) or (**e**,**f**) TAS1R1-TAS1R3 antagonist (gurmarin 30 μg/ml) (n = 9) or their respective vehicle on the effect of (**a**,**c**,**e**) L-Ala 10 mM or (**b**,**d**,**f**) peptone 3% compared to HEPES buffer on octanoyl ghrelin release in the MGN3-1 cells. *P < 0.05, **P < 0.01, ***P < 0.001 vs. vehicle or vehicle with antagonist; ^#^*P* < 0.05, ^##^*P* < 0.01, ^###^*P* < 0.001 in the absence vs. presence of antagonist.

Real-time PCR studies showed that all three known amino acid taste receptors (CaSR, TAS1R1-TAS1R3 and GPRC6A) as well as the peptone receptor, LPAR5, were present in the ghrelinoma cell line although at different expression levels (see Supplementary Fig. S1). The role of these specific amino acid taste receptors in amino acid-induced octanoyl ghrelin release was evaluated for the 3 most potent amino acids (10 mM L-Phe, 10 mM L-Ala, 40 mM MSG) and for 3% peptone. L-Phe-induced octanoyl ghrelin release was significantly (treatment*antagonist P = 0.0004) decreased after preincubation of the cells with the CaSR antagonist, Calhex-231 (5 μM) (Fig. 2a). The TAS1R1-TAS1R3 antagonist, gurmarin, and the GPRC6A antagonist, calindol, were without effect (Fig. 2c,e). The effect of L-Phe was mimicked by the CaSR agonist, cinacalcet (10 μM), which increased octanoyl ghrelin release from 5.99 ± 0.75 to 11.07 ± 2.18 pg/100,000 cells (P = 0.010). The effect of MSG on octanoyl ghrelin release was only reduced by the TAS1R1-TAS1R3 antagonist, gurmarin (30 μg/ml) (treatment*antagonist P = 0.045) (Fig. 2b,d,f). Both calhex-231 (treatment*antagonist P = 0.006) and gurmarin (treatment*antagonist P = 0.02), but not calindol, reduced the effect of L-Ala on octanoyl ghrelin release (Fig. 3a,c,e). Peptone-induced octanoyl ghrelin release involved both the CaSR (treatment*antagonist P = 0.0001) and the GPRC6A (treatment*antagonist P = 0.000006) but not TAS1R1-TAS1R3 (Fig. 3b,d,f).

#### The intracellular Ca^2+^ signalling pathway is involved in MSG- and peptone-induced octanoyl ghrelin release in MGN3-1 cells

**Figure 4:**
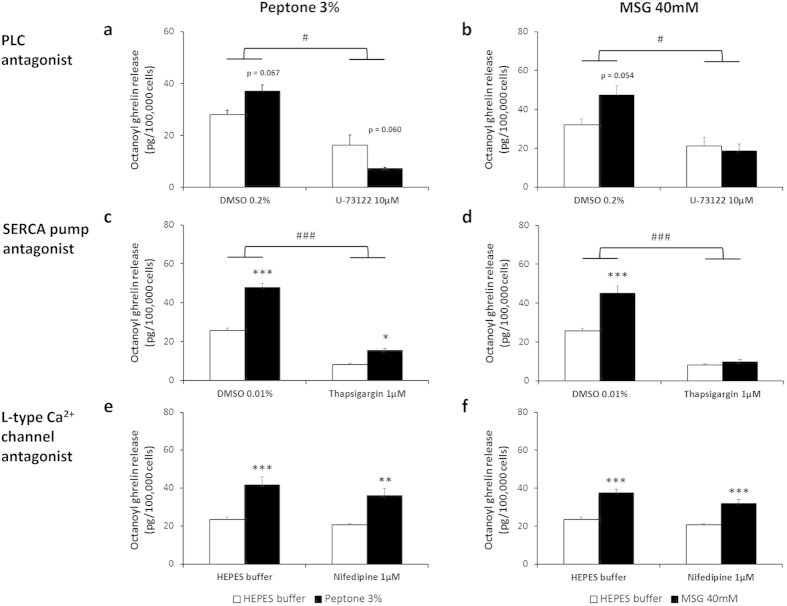
Effect of Ca^2+^-signalling pathway antagonists on peptone- and MSG-induced octanoyl ghrelin release in MGN3-1 cells. Effect of preincubation (30 min) of MGN3-1 cells with a (**a**,**b**) phospholipase C (PLC) antagonist (U-73122 10 μM) (n = 8), (**c**,**d**) SERCA pump antagonist (thapsigargin 1 μM) (n = 6) or (**e**,**f**) L-type Ca^2+^ channel antagonist (nifedipine 1 μM) (n = 6) or their respective vehicle on the effect of (**a**,**c**,**e**) peptone 3% or (**b**,**d**,**f**) MSG 40 mM compared to HEPES buffer on octanoyl ghrelin release in the MGN3-1 cells. *P < 0.05, **P < 0.01, ***P < 0.001 vs. vehicle or vehicle with antagonist; ^#^*P* < 0.05, ^###^*P* < 0.001 in the absence vs. presence of antagonist.

Acute administration of L-Ala (10 mM) or MSG (40 mM) stimulated intracellular Ca^2+^ release in MGN3-1 cells, while L-Phe was without effect at concentrations between 10 and 40 mM (see Supplementary Fig. S2). The acute effect of peptone on intracellular Ca^2+^ release could not be determined due to the inherent yellow colour of peptone which interfered with the fluorescence measurements. However, peptone-induced ghrelin release was blocked after incubation of MGN3-1 cells with the phospholipase C antagonist, U-73122 (10 μM) (treatment*antagonist P = 0.03) and the SERCA pump antagonist, thapsigargin (1 μM) (treatment*antagonist P = 0.00009) but not with the L-type Ca^2+^ channel antagonist, nifedipine (1 μM), thereby confirming a role for intracellular but not extracellular Ca^2+^ (Fig. 4a,c,e). Similar effects were observed with these blockers on MSG-induced ghrelin release (PLC antagonist: treatment*antagonist P = 0.04; SERCA pump blocker: treatment*antagonist P = 0.0004) (Fig. 4b,d,f).

### *Ex vivo* studies in intestinal segments

#### The effect of peptone on octanoyl ghrelin release differs between corpus and jejunum

**Figure 5:**
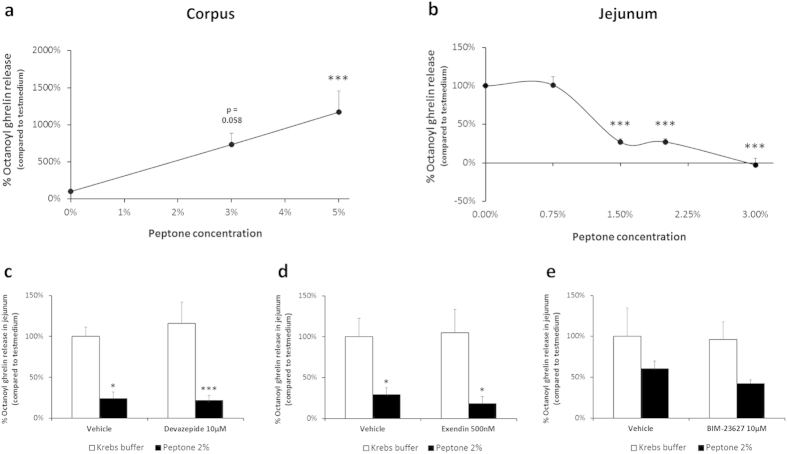
Concentration-dependent effect of peptone on octanoyl ghrelin release in gastric and jejunal mouse tissue segments. Effect of a 2 h stimulation with increasing concentrations of peptone or vehicle (Krebs buffer) on octanoyl ghrelin release from tissue segments of (**a**) the corpus (n = 8–9) or (**b**) the jejunum (n = 3–12). Effect of preincubation (30 min) of jejunal tissue segments with a (**c**) CCK receptor antagonist (devazepide 10 μM) (n 5–6), (**d**) GLP-1 receptor antagonist (exendin (9–39) 500 nM) (n = 5–6) or (**e**) somatostatin receptor antagonist (BIM-23627 10 μM) (n = 5–6) or their respective vehicle on the effect of peptone 2% compared to Krebs buffer on octanoyl ghrelin release from jejunal segments. *P < 0.05, **P < 0.01, ***P < 0.001 vs. vehicle or vehicle with antagonist.

Peptone caused a concentration-dependent increase in octanoyl ghrelin release in segments from the corpus, similar to the effects observed in the gastric ghrelinoma cell line. In contrast, octanoyl ghrelin release was significantly (P = 8·10^−16^) decreased after stimulation of jejunal segments with peptone. Furthermore, the jejunum was more sensitive to peptone since 1.5% peptone decreased octanoyl ghrelin release with 73 ± 4% (P = 0.0006), while in the corpus octanoyl ghrelin release was not yet maximal at 5% peptone (Fig. 5a,b). We hypothesized that the inhibitory effect of peptone on ghrelin release in the jejunum might represent a paracrine effect mediated via peptone-induced release of other hormones (CCK, GLP-1, somatostatin). The role of these three gut peptides in peptone-induced octanoyl ghrelin release was evaluated by pre-treating jejunal segments with a CCK_1_ receptor antagonist (devazepide 10 μM), a GLP-1 receptor antagonist (exendin 9-39 500 nM) or a somatostatin receptor antagonist (BIM-23627 10 μM). However, none of these gut peptide receptor antagonists could reverse the peptone-induced inhibition of octanoyl ghrelin release in the jejunum (Fig. 5c–e).

### *In vivo* studies in mice

#### Intragastric administration of peptone inhibits plasma ghrelin levels

**Figure 6:**
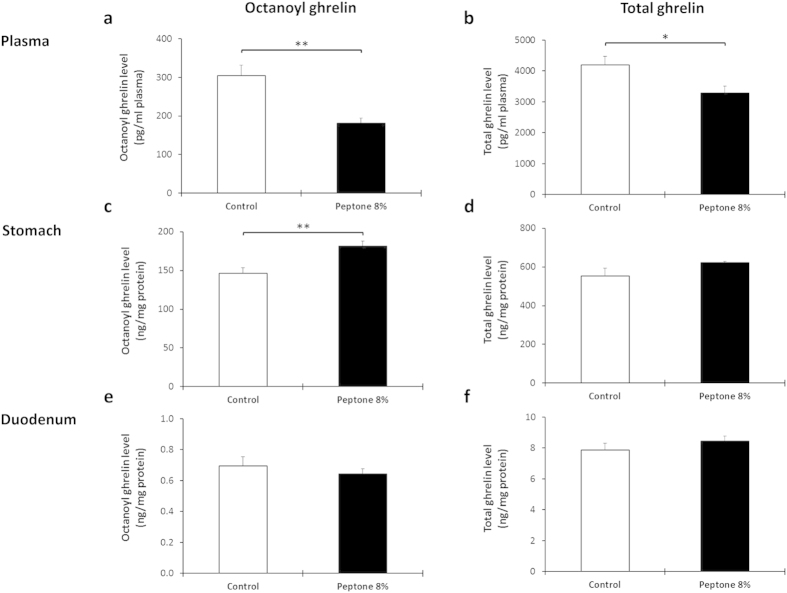
The inhibition in plasma ghrelin levels after intragastric administration of peptone originates mainly from gastric ghrelin cells. Effect of intragastric administration of 8% peptone on octanoyl and total ghrelin secretion. Mice were gavaged with 8% peptone (n = 6–8) or vehicle (water, n = 6–7). Octanoyl and total ghrelin levels were determined in (**a**,**b**) plasma, (**c**,**d**) protein extracts of the stomach and (**e**,**f**) duodenum 40 minutes after injection. *P < 0.05 vs. control; **P < 0.01 vs. control.

Intragastric administration of 8% peptone decreased both octanoyl (P = 0.001) and total plasma ghrelin (P = 0.03) levels with 41 ± 4% and 22 ± 5% respectively (Fig. 6a,b). This was accompanied by a significant increase (P = 0.003) in octanoyl ghrelin content in stomach extracts, but not in duodenal extracts indicating that the inhibition of ghrelin release occurs at the level of the stomach (Fig. 6c–f).

#### Sensing of L-Phe is not polarized

**Figure 7:**
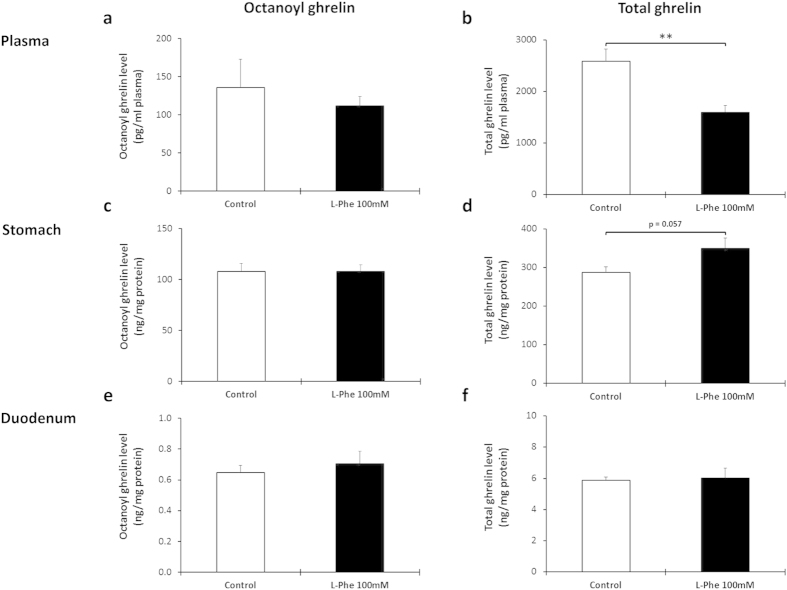
The inhibition in plasma ghrelin levels after intragastric administration of L-Phe originates from gastric ghrelin cells. Effect of intragastric administration of L-Phe on the secretion of octanoyl and total ghrelin. Mice were gavaged with 100 mM L-Phe (n = 6–7) or vehicle (NaCl 0.9%, n = 6–7). Octanoyl and total ghrelin levels were determined in (**a**,**b**) plasma, (**c**,**d**) protein extracts of the stomach and (**e,f**) duodenum 40 minutes after injection. **P < 0.01 vs. control.

**Figure 8:**
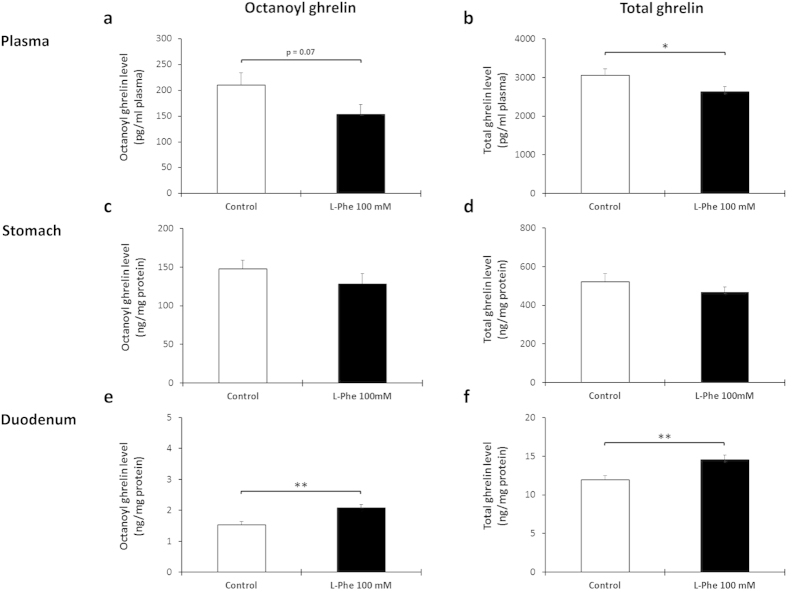
The inhibition in plasma ghrelin levels after intravenous administration of L-Phe originates from duodenal ghrelin cells. Effect of intravenous injection of L-Phe on the secretion of octanoyl and total ghrelin. Mice were intravenously injected into the tail vene with 100 mM L-Phe (n = 9–12) or vehicle (NaCl 0.9%, n = 8–12). Octanoyl and total ghrelin levels were determined in (**a**,**b**) plasma, (**c**,**d**) protein extracts of the stomach and (**e**,**f**) protein extracts of the duodenum 40 minutes after injection. *P < 0.05 vs. control; **P < 0.01 vs. control.

To determine whether L-Phe is sensed by the ghrelin cell in the stomach or duodenum via the lumen or whether amino acid uptake into the blood is required for mediating the effect on ghrelin release, the effect of intragastric versus intravenous administration of L-Phe (100 mM) on ghrelin release was compared. Intragastric administration of L-Phe significantly (P = 0.003) decreased total but not octanoyl plasma ghrelin levels compared to water-treated control mice (Fig. 7a,b). This was probably due to an inhibition of ghrelin release from the stomach since total ghrelin content in the stomach, but not in the duodenum, tended (P = 0.057) to be increased in L-Phe-treated compared to water-treated mice (Fig. 7c–f). Intravenous injection of L-Phe tended (P = 0.07) to decrease octanoyl plasma ghrelin levels and significantly (P = 0.048) decreased total plasma ghrelin levels. This effect was accompanied by an increase in octanoyl (P = 0.003) and total (P = 0.005) ghrelin content in duodenal but not in gastric tissue extracts (Fig. 8).

## Discussion

Our findings in the ghrelinoma cell line MGN3-1 provide for the first time evidence that peptone, umami and specific classes of amino acids (aromatic, aliphatic and sulphur- or hydroxyl-containing) are sensed by amino acid taste receptors (CaSR, GPRC6A, TAS1R1-TAS1R3 or a combination) on the gastric ghrelin cell and stimulate octanoyl ghrelin release. *Ex vivo*, the peptone-induced ghrelin release is mimicked in tissue segments from the mouse corpus, which contain closed-type ghrelin cells, but not from the jejunum, where sensing by open-type ghrelin cells results in an inhibition of ghrelin release. The latter effect is not mediated via an indirect paracrine effect of peptone on nearby GLP-1-, CCK- or somatostatin-containing endocrine cells but probably involves other downstream signalling pathways. In contrast to the *in vitro* and *ex vivo* (corpus tissue) data, intragastric administration of peptone led to a significant reduction in plasma ghrelin levels, originating from a reduced ghrelin release from the gastric but not duodenal ghrelin cells. These findings therefore suggest that *in vivo* an indirect mediator is overriding local nutrient sensing mechanisms. Finally, we observed that amino acid (L-Phe) sensing is not polarized and occurs both via the lumen and the blood stream.

In the first part of this study, we focused on the effect of a casein hydrolysate and of individual amino acids on ghrelin release in the ghrelinoma cell line. Of the 7 different classes of amino acids investigated, the aromatic amino acids (L-Phe and L-Trp), the aliphatic amino acid L-Ala and the sulphur- or hydroxyl-containing amino acids (L-Met and L-Thr) increased octanoyl ghrelin secretion at 10 mM. This concentration falls within the affinity range of amino acid taste receptors for their ligands and is in agreement with the the physiological concentration of free L-amino acids observed in the stomach 3 hours after food intake^17^,^22^,^33^,^34^. Umami stimuli (MSG) affected ghrelin release at higher concentrations (40 mM) and the effect of peptone was maximal at 3%. An amino acid mixture, emulating the fasting levels of amino acids in human plasma, increased octanoyl ghrelin release in MGN3-1 cells, which is in line with the observed increase in octanoyl ghrelin levels in humans during the fasting state^17^.

All putative amino acid taste receptors are expressed in the ghrelinoma cell line. GPRC6A, which is the most sensitive amino acid taste receptor with a binding affinity in the micromolar range^32^, is abundantly expressed whereas expression levels of the putative peptide-responsive receptor LPAR5 were very low. Our studies showed that sensing of L-Phe occurs via the CaSR, MSG via the TAS1R1-TAS1R3 while L-Ala and peptone act via 2 different amino acid taste receptors: CaSR & TAS1R1-TAS1R3 and CaSR & GPRC6A, respectively. The involvement of multiple receptors for amino acid sensing was demonstrated before both in the lingual epithelium and in the gut^35^,^36^. CCK secretion following L-Phe administration in mouse proximal small intestine segments involved both the CaSR and the TAS1R1-TAS1R3^35^.

Until now, the CaSR is involved in L-Phe-induced release of four other gut peptides: gastrin and somatostatin in the stomach and CCK and GLP-1 in the small intestine. Studies in wildtype and CaSR^−/−^ mice confirmed that the CaSR is mediating the effect of oral administration of L-Phe on gastrin release^37^. Immunohistochemistry studies showed that a small population of CaSR-containing cells in the antrum of human, mouse and pig also express somatostatin^18^. Two independent studies demonstrated that the CaSR is involved in sensing of L-Phe both in the CCK-producing enteroendocrine cell line, STC-1, and in native I-cells^19^,^38^. In humans, administration of L-Phe before a meal enhanced CCK secretion and decreased subsequent food intake^39^. Finally, perfusion of isolated loops of rat small intestine with L-Phe stimulated GLP-1 secretion via the CaSR^40^. The role of CaSR in the release of ghrelin is less straightforward. Engelstoft *et al.* (2013) observed a concentration-dependent decrease in octanoyl ghrelin release in primary gastric ghrelin cells after administration of R-568, an allosteric agonist of the CaSR in the presence of 1.8 mM CaCl_2_. Increasing the CaCl_2_ concentration to 4 mM shifted the effect of R-568 on ghrelin release from an inhibition to a stimulation^41^. In contrast, we showed an increase in octanoyl ghrelin release after stimulation with the calcimimetic agent cinacalcet in the presence of 1.8 mM CaCl_2_.

Both the CaSR and GPRC6A, known to be involved in amino acid-induced GLP-1 release from GLUTag cells, mediate the effect of peptone on ghrelin release^42^. The low expression level of the putative peptone receptor, LPAR5, did not allow us to perform reproducible and effective RNA silencing studies. Therefore the role of this receptor remains elusive.

Gurmarin, an inhibitor of the sweet taste receptor complex TAS1R2-TAS1R3, also inhibits sensing of TAS1R1-TAS1R3 through binding to the extracellular venus flytrap module of TAS1R3 leading to suppressed umami responses in taste buds^43^. Gurmarin partially reduced the increased octanoyl ghrelin secretion in response to MSG in the ghrelinoma cell line.

Our observation that both MSG and L-Ala activate a common taste receptor (TAS1R1-TAS1R3) is supported by studies in the lingual system and the finding that these 2 amino acids share perceptual qualities in rats^44^. In addition, L-Ala-induced ghrelin release is also mediated by the CaSR. Previous studies showed that L-Ala is sensed by the CaSR in HEK-293 cells expressing the human CaSR and in normal human parathyroid cells^17^,^45^.

The intracellular signalling pathways involved in amino acid sensing by specific taste receptors are mainly studied in the tongue (recently reviewed^46^,^47^) but similar studies in enteroendocrine cells are emerging. Intracellular Ca^2+^ mobilization plays a key role in amino acid sensing by GPRC6A in GLUTag cells and by CaSR in STC-1 cells leading to GLP-1 and CCK secretion, respectively^38^,^42^. In the present study, a role for the intracellular Ca^2+^ signalling pathway was revealed in the effect of L-Ala, MSG and peptone but not of L-Phe on ghrelin release. It is therefore likely that both TAS1R1-TAS1R3 and GPRC6A, involved in sensing of L-Ala and MSG, but not CaSR, mediating the effect of L-Phe, are predominantly Gα_q_-coupled. Studies in isolated primary ghrelin cells and in CaSR-transfected HEK-293 cells showed that CaSR has the potential for biased signalling and couples through both Gα_q_ and Gα_i_^41^,^48^. At least in the ghrelinoma cell line, intracellular Ca^2+^ does not seem to be mobilized after activation of CaSR. It is therefore likely that CaSR is coupled to Gα_s_ as has been reported for AtT-20 cells^49^.

In the second part of the study, the effect of peptone on ghrelin release was studied in gastric and jejunal segments, which represent a more integrated system. In addition this enabled us to compare whether the functional responses of the closed-type ghrelin cells in the stomach differ from the opened-type ghrelin cells that are in contact with the lumen in the small intestine^31^. Peptone induced a concentration-dependent increase in octanoyl ghrelin release from tissue segments of the corpus, thereby mimicking the observed effect in the ghrelinoma cell line, originating from a gastric ghrelin-producing tumour. In contrast, a concentration-dependent decrease in octanoyl ghrelin release was observed in jejunal segments. Since peptone is known to stimulate the release of gut peptide hormones CCK, GLP-1 and somatostatin, which can in turn inhibit ghrelin release, we hypothesized that the inhibitory effect of peptone on ghrelin release in jejunal segments was indirect and mediated via peptone-induced release of these gut hormones^50^,^51^,^52^. However, none of the receptor antagonists for these hormones could reverse the peptone-induced ghrelin inhibition. In line with our observations, GLP-1 or CCK did not induce octanoyl ghrelin release in isolated primary ghrelin cells which expressed the GLP-1 and both the CCK_1_ and CCK_2_ receptor^41^. Nevertheless, three out of five somatostatin receptors (SSTR1, SSTR2, SSTR3) are expressed at relatively high levels in primary ghrelin cells but somatostatin only reduced ghrelin secretion partially^41^. We speculate that other downstream signalling pathways are coupled to the taste receptors in the jejunum, resulting in inhibition of ghrelin release but future studies are necessary to elucidate these mechanisms.

Intragastric administration of 8% peptone in mice decreased plasma octanoyl and total ghrelin levels, which was due to an inhibition of octanoyl ghrelin release from the stomach. Indeed, in humans, others described a reduction in ghrelin release after a casein-rich meal^53^. These observations are in line with the physiological function of ghrelin as an orexigenic or meal initiating factor. In humans plasma ghrelin levels decrease after a meal, mimicked in the present study by peptone, to prevent further food intake and increase thereafter to dictate the timing of the next meal^54^. Nevertheless, these *in vivo* results are at variance with the increase in octanoyl ghrelin release in the ghrelinoma cell line and in the gastric mucosal segments after stimulation with peptone, suggesting that these local effects are overruled *in vivo* by an indirect mechanism. Our studies with jejunal segments together with the findings in primary gastric ghrelin cells argue against an important role of GLP-1, CCK or somatostatin.

It is still possible that the inhibition of gastric ghrelin release after intragastric administration with peptone originates from intestinal regions more distally from the stomach and the jejunum. A possible candidate is PYY, mainly secreted by the L-cells in the distal gut. Ullrich *et al.* (2015) showed that intraduodenal administration of a whey hydrolysate in humans led to reduced plasma ghrelin levels accompanied by an increase in PYY levels^55^. Although this suggests that PPY can function as a regulator of ghrelin secretion, Engelstoft *et al.* (2013) could not demonstrate the presence of the NPY1 or NPY2 receptor on isolated ghrelin cells^41^.

Knowing that ghrelin and insulin levels correlate inversely after intake of a protein-rich meal in humans and that a casein hydrolysate is able to stimulate insulin release, insulin is also a possible candidate for the direct inhibitory effect of peptone on ghrelin release^56^,^57^. The insulin receptor is expressed in the ghrelinoma cell line and in a primary culture of gastric ghrelin-secreting cells^58^,^59^. However the effects of insulin on ghrelin release in these cells are contradictory: two studies reported a decrease, while two other studies did not observe an effect^58^,^59^,^60^,^61^. We could also not confirm an effect of insulin on ghrelin release in the ghrelinoma cell line (data not shown). Moreover the observed peptone-induced decrease in octanoyl ghrelin release in the jejunal segments cannot be clarified by the effect of insulin.

A potential role for the enteric nervous system (ENS) or the vagus in the effect of amino acids on ghrelin release *in vivo* cannot be excluded. Amino acids/peptides might be sensed by the enteric nerve endings after absorption. It is known that the dipeptide receptor PEPT2 and amino acid receptors involved in glutamate and glycine sensing are present on enteric neurons^62^,^63^,^64^ which directly increase the excitability of the enteric neurons^63^,^65^,^66^. Perhaps more importantly, amino acids affect the synthesis and release of neurotransmitters that can initiate a neural reflex to affect ghrelin release. L-cysteine and L-arginine, for example, are substrates for enzymes involved in the synthesis of the enteric neurotransmitters, nitric oxide and hydrogen sulfide, respectively^67^. Uptake of L-tryptophan can increase the synthesis and release of serotonin since tryptophan hydroxylase 2 (TPH2), the enzyme responsible for the production of 5-HT, is produced by enteric neurons^68^.

In addition, administration of amino acids (L-Lysine and MSG) or a meat hydrolysate activate vagal afferent fibers, indicating that the vagus nerve is of importance to transfer peripheral information to the central nervous system after amino acid/peptide intake^69^,^70^,^71^,^72^. Several *in vivo* studies indicate that the fasting-induced increase in plasma ghrelin levels is mediated by both the cholinergic and adrenergic arms of the autonomic nervous system^73^. Although the presence of β1 adrenergic receptors has been confirmed on the ghrelin cell, acetylcholine fails to affect ghrelin release in MGN3-1 ghrelinoma cells or primary cultures from rat stomach^58^,^74^,^75^. However, further research is required to clarify the role of the ENS and vagus nerve in the effect of amino acids on ghrelin release.

Since amino acids can be absorbed via various apical amino acid transporters with certain group specificities, we determined whether L-Phe is sensed via the luminal or blood-born direction^76^. Both intragastric and intravenous administration of L-Phe significantly decreased plasma total ghrelin levels while the effect on octanoyl ghrelin release was less pronounced. Thus overall sensing of L-Phe is not polarized although the sensing in the different regions of the gut seems to be dependent upon the route of administration. The ghrelin content in the duodenum was significantly increased after intravenous administration whereas the ghrelin content in the stomach was significantly increased after intragastric administration. These findings highlight the complexity of the mechanisms in the postprandial effects of ghrelin.

In conclusion, amino acids and di-/tripeptides are sensed by various taste receptors on the ghrelin cells that stimulate a chemosensory signalling pathway regulating local ghrelin release which might be stimulatory or inhibitory depending on the region in the gut. *In vivo,* post-absorptive and indirect mechanisms, probably involving insulin or another signal, overrule these effects to shut down the release of ghrelin and hence hunger signalling.

## Materials and Methods

### Materials

All amino acids and peptone were obtained from Sigma (St. Louis, MO). The composition of the amino acid mixture mimicking the fasting levels of amino acids in human plasma^17^ (referred to as L-AA mix) was as follows (in μM): 50 L-Phe; 50 L-Trp; 80 L-His; 60 L-Tyr; 30 L-Cys; 300 L-Ala; 200 L-Thr; 50 L-Asn; 600 L-Gln; 125 L-Ser; 30 L-Glu; 250 L-Gly; 180 L-Pro; 250 L-Val; 30 L-Met; 10 L-Asp; 200 L-Lys; 100 L-Arg; 75 L-Ile; 150 L-Leu. The CaSR agonist, cinacalcet, was obtained from Biorbyt (cinacalcet hydrochloride, Biorbyt Ltd., Cambridge, United Kingdom). The CaSR antagonist, calhex-231, and the GPRC6A antagonist, calindol, were purchased from Santa Cruz Biotechnology (Santa Cruz Biotechnology, Inc., Dallas, Texas). All stock solutions were made in DMSO and further dilutions were made in HEPES buffer resulting in a final concentration of 0.1% DMSO (CaSR agonist), 0.01% DMSO (CaSR antagonist) and 0.01% DMSO (GPRC6A antagonist) during the cell experiment. Gurmarin was heterologously secreted using the yeast *Pichia pastoris* and subsequently purified as previously described^77^.

### Mice

Male C57BL/6 WT mice were obtained from Janvier. All mice (11–12 weeks of age) were housed (20–22 °C) under a 14-h:10-h light-dark cycle and had ad libitum access to food and drinking water. All experimental procedures were approved and carried out in accordance to the regulations and guidelines of the Ethical committee for Animal Experiments of the KU Leuven (project number 048/2011).

### Experimental design

Overnight-fasted mice were either gavaged with peptone 8%, L-Phe 100 mM or vehicle or injected intravenously (IV) into the tail vene with 100 mM L-Phe or vehicle. Forty minutes after IV injection or gavage, mice were sacrificed and blood was collected by cardiac puncture. The stomach and duodenum were removed and stored for protein extraction.

### Ghrelin tissue extraction

Tissue from stomach and duodenum was boiled for 10 minutes followed by homogenization in 3 volumes of water with protease inhibitors (MP Biomedicals, Santa Ana, CA) and 9 volumes of 6% acetic acid. After 10 minutes of boiling, the homogenate was centrifuged to collect the supernatant which was diluted and subjected to radioimmunoassay (RIA). Protein levels were determined using the Pierce BCA Protein Assay Kit (Thermo Fisher Scientific Inc., Waltham, MA).

### Ghrelin release from ghrelinoma cells

The ghrelinoma cell line, MGN3-1, was kindly provided by Prof. Hiroshi Iwakura (Kyoto University Hospital, Kyoto, Japan). Cells were cultured in Dulbecco’s modified eagle medium (DMEM) supplemented with 10% fetal bovine serum (FBS) and 1% penicillin and streptomycin at 5% CO_2_ and at 37 °C. Cells were incubated for 3 hours with the individual amino acids, L-AA mix, peptone or cinacalcet^78^ at the indicated concentration in HEPES buffer. The effect of specific amino acid taste receptor antagonists (calhex-231 5 μM^40^,^79^, calindol 5 μM^42^,^80^ or gurmarin 30 ng/mg^35^,^77^) and Ca^2+^ signalling pathway antagonists (U-73122 10 μM^42^,^81^, thapsigargin 1 μM^58^,^82^ or nifedipine 1 μM^83^) was investigated by preincubation of the cells during 30 minutes with the respective antagonist after which the culture medium was removed and replaced by a combination of the antagonist and an amino acid or peptone. Following 3 hours of incubation, the supernatant was collected, acidified (0.1M HCl) and stored at −80 °C.

### Ghrelin release from intestinal segments

Overnight fasted mice were refed for 2 hours prior to being sacrificed. Segments of the intact corpus of the stomach (0.2 × 0.2 cm) and jejunum (0.2 × 0.5 cm) were dissected and incubated at 37 °C in Krebs buffer with the test solutions for 2 hours. The culture medium was collected, acidified (1M HCl) and stored at −80 °C for RIA. The role of GLP-1, CCK and somatostatin was investigated by preincubation of the intact jejunum (0.2 × 0.8 cm) during 30 minutes with the respective antagonist (exendin (9–39) 500 nM^84^,^85^, devazepide 10 μM^86^,^87^, BIM-23627 10 μM^41^,^88^) after which the culture medium was removed and replaced by a combination of the antagonist and peptone. Following 2 hours of incubation, the supernatant was collected, acidified (0.1 M HCl) and stored at −80 °C. Tissue segments were dried to correct the ghrelin release for dry tissue weight of the segment.

### Radioimmunoassay (RIA)

Plasma samples and cell/tissue culture supernatants were extracted on a SEP-Pak C18 cartridge (Waters Corporation, Milford, MA), vacuum-dried and subjected to ghrelin RIA. The RIA was performed as previously described^29^.

### Intracellular Ca^2+^ release experiments

MGN3-1 cells were loaded with 10 μM Fluo-4-AM (Molecular Probes, Life Technologies, Paisley, UK) at room temperature in HEPES buffer (pH 7.4) during 20 minutes. Changes in intracellular Ca^2+^ release [Ca^2+^]_i_ were measured in a fluorescence reader (FLUOstar Omega, BMG Laboratories, Offenburg, Germany) at 520 nm and normalized to their initial baseline value recorded 1 min before the application of the test compound.

### Quantitative real-time PCR

RNA was isolated from MGN3-1 cells using the Qiagen RNeasy kit (Qiagen, Hilden, Germany) and reversed transcribed to cDNA using Superscript II Reverse Transcriptase (Invitrogen, Carlsbad, CA, USA). Quantitative real-time PCR was performed as previously described^89^. Primer sequences were as follows: GAPDH: forward CCCCAATgTgTCCgTCgTg, reverse gCCTgCTTCACCACCTTCT; TAS1R1: forward CCCCTggCAgCTTCTTCAgCAg, reverse AggTCCATTCCAgTCCCAggCg; TAS1R3: forward CCCAACAgCATCCCgTgCAA, reverse CTCCACAgCCATCTTCATAgC; CaSR: forward ACCTgCTTACCCggAAgAgggCTTT, reverse AATTCAggTgCCgTAggTgTTTCAg; GPRC6A: forward gTgTTCgCCCTTggTCATgCCA, reverse CAgCACggCAAgTAgCTCCCATg; LPAR5: forward TCCACgCTggCTgTATATgg, reverse TCgCggTCCTgAATACTgTTC.

### Statistical analysis

Results are presented as means ± SEM. Data were analysed using Statistica 12 (StatSoft Inc., Tulsa, OK, USA). Differences in octanoyl ghrelin release in the presence of individual amino acids/the amino acid mixture compared to the control stimulation in MGN3-1 cells and the difference between peptone-/L-Phe-treated mice and vehicle-treated mice in gavage and IV experiments were analysed with an unpaired Student’s t-test. All other data were analysed using a one- or two-way ANOVA (factors: treatment and antagonists). In case of a significant interaction effect or treatment effect, tests with contrasts were performed to locate pairs of factor levels with significant differences in the examined variables followed by post hoc Bonferroni testing. Significance was accepted at the 5% level.

## Additional Information

**How to cite this article**: Vancleef, L. *et al.* Chemosensory signalling pathways involved in sensing of amino acids by the ghrelin cell. *Sci. Rep.* **5**, 15725; doi: 10.1038/srep15725 (2015).

## Supplementary Material

Supplementary Information

## References

[b1] Sjostrom L. *et al.* Effects of bariatric surgery on mortality in Swedish obese subjects. N. Engl. J. Med. 357, 741–752 (2007). 17715408 10.1056/NEJMoa066254

[b2] Mingrone G. *et al.* Bariatric surgery versus conventional medical therapy for type 2 diabetes. N. Engl. J. Med. 366, 1577–1585 (2012). 22449317 10.1056/NEJMoa1200111

[b3] Westerterp-Plantenga M. S. The significance of protein in food intake and body weight regulation. Curr. Opin. Clin. Nutr. Metab. Care. 6, 635–638 (2003). 14557793 10.1097/00075197-200311000-00005

[b4] Larsen T. M. *et al.* Diets with high or low protein content and glycemic index for weight-loss maintenance. N. Engl. J. Med. 363, 2102–2113 (2010). 21105792 10.1056/NEJMoa1007137 PMC3359496

[b5] Westerterp-Plantenga M. S., Nieuwenhuizen A., Tome D., Soenen S. & Westerterp K. R. Dietary protein, weight loss, and weight maintenance. Annu. Rev. Nutr. 29, 21–41 (2009). 19400750 10.1146/annurev-nutr-080508-141056

[b6] Batterham R. L. *et al.* Critical role for peptide YY in protein-mediated satiation and body-weight regulation. Cell Metab. 4, 223–233 (2006). 16950139 10.1016/j.cmet.2006.08.001

[b7] Lejeune M. P., Westerterp K. R., Adam T. C., Luscombe-Marsh N. D. & Westerterp-Plantenga M. S. Ghrelin and glucagon-like peptide 1 concentrations, 24-h satiety, and energy and substrate metabolism during a high-protein diet and measured in a respiration chamber. Am. J. Clin. Nutr. 83, 89–94 (2006). 16400055 10.1093/ajcn/83.1.89

[b8] Smeets A. J., Soenen S., Luscombe-Marsh N. D., Ueland O. & Westerterp-Plantenga M. S. Energy expenditure, satiety, and plasma ghrelin, glucagon-like peptide 1, and peptide tyrosine-tyrosine concentrations following a single high-protein lunch. J. Nutr. 138, 698–702 (2008). 18356323 10.1093/jn/138.4.698

[b9] Kojima M. *et al.* Ghrelin is a growth-hormone-releasing acylated peptide from stomach. Nature. 402, 656–660 (1999). 10604470 10.1038/45230

[b10] Gutierrez J. A. *et al.* Ghrelin octanoylation mediated by an orphan lipid transferase. Proc. Natl. Acad. Sci. USA 105, 6320–6325 (2008). 18443287 10.1073/pnas.0800708105 PMC2359796

[b11] Yang J., Brown M. S., Liang G., Grishin N. V. & Goldstein J. L. Identification of the acyltransferase that octanoylates ghrelin, an appetite-stimulating peptide hormone. Cell. 132, 387–396 (2008). 18267071 10.1016/j.cell.2008.01.017

[b12] Depoortere I. Targeting the ghrelin receptor to regulate food intake. Regul. Pept. 156, 13–23 (2009). 19362579 10.1016/j.regpep.2009.04.002

[b13] Avau B., Carbone F., Tack J. & Depoortere I. Ghrelin signaling in the gut, its physiological properties, and therapeutic potential. Neurogastroenterol. Motil. 25, 720–732 (2013). 23910374 10.1111/nmo.12193

[b14] Verhulst P. J. & Depoortere I. Ghrelin’s second life: from appetite stimulator to glucose regulator. World J. Gastroenterol. 18, 3183–3195 (2012). 22783041 10.3748/wjg.v18.i25.3183 PMC3391754

[b15] Janssen S. & Depoortere I. Nutrient sensing in the gut: new roads to therapeutics? Trends Endocrinol. Metab. 24, 92–100 (2013). 23266105 10.1016/j.tem.2012.11.006

[b16] Depoortere I. Taste receptors of the gut: emerging roles in health and disease. Gut. 63, 179–190 (2014). 24131638 10.1136/gutjnl-2013-305112

[b17] Conigrave A. D., Quinn S. J. & Brown E. M. L-amino acid sensing by the extracellular Ca2+-sensing receptor. Proc. Natl. Acad. Sci. USA 97, 4814–4819 (2000). 10781086 10.1073/pnas.97.9.4814 PMC18315

[b18] Haid D. C., Jordan-Biegger C., Widmayer P. & Breer H. Receptors responsive to protein breakdown products in g-cells and d-cells of mouse, swine and human. Front. Physiol. 3, 65 (2012). 22514536 10.3389/fphys.2012.00065 PMC3322525

[b19] Liou A. P. *et al.* The extracellular calcium-sensing receptor is required for cholecystokinin secretion in response to L-phenylalanine in acutely isolated intestinal I cells. Am. J. Physiol. Gastrointest. Liver Physiol. 300, G538–546 (2011). 21252045 10.1152/ajpgi.00342.2010 PMC3074990

[b20] Wellendorph P., Johansen L. D. & Brauner-Osborne H. Molecular pharmacology of promiscuous seven transmembrane receptors sensing organic nutrients. Mol. Pharmacol. 76, 453–465 (2009). 19487246 10.1124/mol.109.055244

[b21] Li X. *et al.* Human receptors for sweet and umami taste. Proc. Natl. Acad. Sci. USA 99, 4692–4696 (2002). 11917125 10.1073/pnas.072090199 PMC123709

[b22] Nelson G. *et al.* An amino-acid taste receptor. Nature. 416, 199–202 (2002). 11894099 10.1038/nature726

[b23] Hass N., Schwarzenbacher K. & Breer H. T1R3 is expressed in brush cells and ghrelin-producing cells of murine stomach. Cell Tissue Res. 339, 493–504 (2010). 20063013 10.1007/s00441-009-0907-6

[b24] Jang H. J. *et al.* Gut-expressed gustducin and taste receptors regulate secretion of glucagon-like peptide-1. Proc. Natl. Acad. Sci. USA 104, 15069–15074 (2007). 17724330 10.1073/pnas.0706890104 PMC1986614

[b25] Akiba Y., Watanabe C., Mizumori M. & Kaunitz J. D. Luminal L-glutamate enhances duodenal mucosal defense mechanisms via multiple glutamate receptors in rats. Am. J. Physiol. Gastrointest. Liver Physiol. 297, G781–791 (2009). 19643955 10.1152/ajpgi.90605.2008 PMC2763813

[b26] Choi S., Lee M., Shiu A. L., Yo S. J. & Aponte G. W. Identification of a protein hydrolysate responsive G protein-coupled receptor in enterocytes. Am. J. Physiol. Gastrointest. Liver Physiol. 292, G98–G112 (2007). 16935853 10.1152/ajpgi.00295.2006

[b27] Hundal H. S. & Taylor P. M. Amino acid transceptors: gate keepers of nutrient exchange and regulators of nutrient signaling. Am. J. Physiol. Endocrinol. Metab. 296, E603–613 (2009). 19158318 10.1152/ajpendo.91002.2008 PMC2670634

[b28] Nassl A. M., Rubio-Aliaga I., Sailer M. & Daniel H. The intestinal peptide transporter PEPT1 is involved in food intake regulation in mice fed a high-protein diet. PLoS One. 6, e26407 (2011). 22031831 10.1371/journal.pone.0026407 PMC3198773

[b29] Janssen S. *et al.* Bitter taste receptors and alpha-gustducin regulate the secretion of ghrelin with functional effects on food intake and gastric emptying. Proc. Natl. Acad. Sci. USA 108, 2094–2099 (2011). 21245306 10.1073/pnas.1011508108 PMC3033292

[b30] Janssen S., Laermans J., Iwakura H., Tack J. & Depoortere I. Sensing of fatty acids for octanoylation of ghrelin involves a gustatory G-protein. PLoS One. 7, e40168 (2012). 22768248 10.1371/journal.pone.0040168 PMC3387020

[b31] Sakata I. *et al.* Ghrelin-producing cells exist as two types of cells, closed- and opened-type cells, in the rat gastrointestinal tract. Peptides. 23, 531–536 (2002). 11836003 10.1016/s0196-9781(01)00633-7

[b32] Wellendorph P. *et al.* Deorphanization of GPRC6A: a promiscuous L-alpha-amino acid receptor with preference for basic amino acids. Mol. Pharmacol. 67, 589–597 (2005). 15576628 10.1124/mol.104.007559

[b33] Christiansen B., Hansen K. B., Wellendorph P. & Brauner-Osborne H. Pharmacological characterization of mouse GPRC6A, an L-alpha-amino-acid receptor modulated by divalent cations. Br. J. Pharmacol. 150, 798–807 (2007). 17245368 10.1038/sj.bjp.0707121 PMC2013871

[b34] Adibi S. A. & Mercer D. W. Protein digestion in human intestine as reflected in luminal, mucosal, and plasma amino acid concentrations after meals. J. Clin. Invest. 52, 1586–1594 (1973). 4718954 10.1172/JCI107335 PMC302429

[b35] Daly K. *et al.* Sensing of amino acids by the gut-expressed taste receptor T1R1-T1R3 stimulates CCK secretion. Am. J. Physiol. Gastrointest. Liver Physiol. 304, G271–282 (2013). 23203156 10.1152/ajpgi.00074.2012 PMC3566511

[b36] Yasumatsu K. *et al.* Umami taste in mice uses multiple receptors and transduction pathways. J. Physiol. 590, 1155–1170 (2012). 22183726 10.1113/jphysiol.2011.211920 PMC3381822

[b37] Feng J. *et al.* Calcium-sensing receptor is a physiologic multimodal chemosensor regulating gastric G-cell growth and gastrin secretion. Proc. Natl. Acad. Sci. USA 107, 17791–17796 (2010). 20876097 10.1073/pnas.1009078107 PMC2955134

[b38] Hira T., Nakajima S., Eto Y. & Hara H. Calcium-sensing receptor mediates phenylalanine-induced cholecystokinin secretion in enteroendocrine STC-1 cells. FEBS J. 275, 4620–4626 (2008). 18691347 10.1111/j.1742-4658.2008.06604.x

[b39] Ballinger A. B. & Clark M. L. L-phenylalanine releases cholecystokinin (CCK) and is associated with reduced food intake in humans: evidence for a physiological role of CCK in control of eating. Metabolism. 43, 735–738 (1994). 8201963 10.1016/0026-0495(94)90123-6

[b40] Mace O. J., Schindler M. & Patel S. The regulation of K- and L-cell activity by GLUT2 and the calcium-sensing receptor CasR in rat small intestine. J. Physiol. 590, 2917–2936 (2012). 22495587 10.1113/jphysiol.2011.223800 PMC3448156

[b41] Engelstoft M. S. *et al.* Seven transmembrane G protein-coupled receptor repertoire of gastric ghrelin cells. Mol. Metab. 2, 376–392 (2013). 24327954 10.1016/j.molmet.2013.08.006 PMC3854997

[b42] Oya M. *et al.* The G protein-coupled receptor family C group 6 subtype A (GPRC6A) receptor is involved in amino acid-induced glucagon-like peptide-1 secretion from GLUTag cells. J. Biol. Chem. 288, 4513–4521 (2013). 23269670 10.1074/jbc.M112.402677 PMC3576058

[b43] Sigoillot M., Brockhoff A., Meyerhof W. & Briand L. Sweet-taste-suppressing compounds: current knowledge and perspectives of application. Appl. Microbiol. Biotechnol. 96, 619–630 (2012). 22983596 10.1007/s00253-012-4387-3

[b44] Taylor-Burds C. C., Westburg A. M., Wifall T. C. & Delay E. R. Behavioral comparisons of the tastes of L-alanine and monosodium glutamate in rats. Chem. Senses. 29, 807–814 (2004). 15574816 10.1093/chemse/bjh246

[b45] Conigrave A. D. *et al.* L-amino acids regulate parathyroid hormone secretion. J. Biol. Chem. 279, 38151–38159 (2004). 15234970 10.1074/jbc.M406373200

[b46] Brennan S. C., Davies T. S., Schepelmann M. & Riccardi D. Emerging roles of the extracellular calcium-sensing receptor in nutrient sensing: control of taste modulation and intestinal hormone secretion. Br. J. Nutr. 111 Suppl 1, S16–22 (2014). 24382107 10.1017/S0007114513002250

[b47] Yasuo T., Kusuhara Y., Yasumatsu K. & Ninomiya Y. Multiple receptor systems for glutamate detection in the taste organ. Biol. Pharm. Bull. 31, 1833–1837 (2008). 18827337 10.1248/bpb.31.1833

[b48] Davey A. E. *et al.* Positive and negative allosteric modulators promote biased signaling at the calcium-sensing receptor. Endocrinology. 153, 1232–1241 (2012). 22210744 10.1210/en.2011-1426

[b49] Mamillapalli R. & Wysolmerski J. The calcium-sensing receptor couples to Galpha(s) and regulates PTHrP and ACTH secretion in pituitary cells. J. Endocrinol. 204, 287–297 (2010). 20032198 10.1677/JOE-09-0183 PMC3777408

[b50] Calbet J. A. & Holst J. J. Gastric emptying, gastric secretion and enterogastrone response after administration of milk proteins or their peptide hydrolysates in humans. Eur. J. Nutr. 43, 127–139 (2004). 15168035 10.1007/s00394-004-0448-4

[b51] Chayvialle J. A., Miyata M., Rayford P. L. & Thompson J. C. Effects of test meal, intragastric nutrients, and intraduodenal bile on plasma concentrations of immunoreactive somatostatin and vasoactive intestinal peptide in dogs. Gastroenterology. 79, 844–852 (1980). 6106620

[b52] Nakajima S., Hira T. & Hara H. Calcium-sensing receptor mediates dietary peptide-induced CCK secretion in enteroendocrine STC-1 cells. Mol. Nutr. Food Res. 56, 753–760 (2012). 22648622 10.1002/mnfr.201100666

[b53] Veldhorst M. A. *et al.* Dose-dependent satiating effect of whey relative to casein or soy. Physiol. Behav. 96, 675–682 (2009). 19385022 10.1016/j.physbeh.2009.01.004

[b54] Cummings D. E. *et al.* A preprandial rise in plasma ghrelin levels suggests a role in meal initiation in humans. Diabetes. 50, 1714–1719 (2001). 11473029 10.2337/diabetes.50.8.1714

[b55] Ullrich S. S. *et al.* Comparative effects of intraduodenal protein and lipid on ghrelin, peptide YY, and leptin release in healthy men. Am. J. Physiol. Regul. Integr. Comp. Physiol. 308, R300–304 (2015). 25568079 10.1152/ajpregu.00504.2014

[b56] Foster-Schubert K. E. *et al.* Acyl and total ghrelin are suppressed strongly by ingested proteins, weakly by lipids, and biphasically by carbohydrates. J. Clin. Endocrinol. Metab. 93, 1971–1979 (2008). 18198223 10.1210/jc.2007-2289 PMC2386677

[b57] Jonker J. T. *et al.* Effects of low doses of casein hydrolysate on post-challenge glucose and insulin levels. Eur. J. Intern. Med. 22, 245–248 (2011). 21570642 10.1016/j.ejim.2010.12.015

[b58] Gagnon J. & Anini Y. Insulin and norepinephrine regulate ghrelin secretion from a rat primary stomach cell culture. Endocrinology. 153, 3646–3656 (2012). 22691550 10.1210/en.2012-1040

[b59] Iwakura H. *et al.* Establishment of a novel ghrelin-producing cell line. Endocrinology. 151, 2940–2945 (2010). 20375182 10.1210/en.2010-0090

[b60] Zhao T. J. *et al.* Ghrelin O-acyltransferase (GOAT) is essential for growth hormone-mediated survival of calorie-restricted mice. Proc. Natl. Acad. Sci. USA 107, 7467–7472 (2010). 20231469 10.1073/pnas.1002271107 PMC2867684

[b61] Sakata I. *et al.* Glucose-mediated control of ghrelin release from primary cultures of gastric mucosal cells. Am. J. Physiol. Endocrinol. Metab. 302, E1300–1310 (2012). 22414807 10.1152/ajpendo.00041.2012 PMC3361986

[b62] Galligan J. J. Ligand-gated ion channels in the enteric nervous system. Neurogastroenterol. Motil. 14, 611–623 (2002). 12464083 10.1046/j.1365-2982.2002.00363.x

[b63] Neunlist M. *et al.* Glycine activates myenteric neurones in adult guinea-pigs. J. Physiol. 536, 727–739 (2001). 11691868 10.1111/j.1469-7793.2001.00727.x PMC2278892

[b64] Ruhl A., Hoppe S., Frey I., Daniel H. & Schemann M. Functional expression of the peptide transporter PEPT2 in the mammalian enteric nervous system. J. Comp. Neurol. 490, 1–11 (2005). 16041713 10.1002/cne.20617

[b65] Cherubini E. & North R. A. Actions of gamma-aminobutyric acid on neurones of guinea-pig myenteric plexus. Br. J. Pharmacol. 82, 93–100 (1984). 6733360 10.1111/j.1476-5381.1984.tb16445.x PMC1987234

[b66] Liu M. T., Rothstein J. D., Gershon M. D. & Kirchgessner A. L. Glutamatergic enteric neurons. J. Neurosci. 17, 4764–4784 (1997). 9169536 10.1523/JNEUROSCI.17-12-04764.1997 PMC6573355

[b67] Neunlist M. & Schemann M. Nutrient-induced changes in the phenotype and function of the enteric nervous system. J. Physiol. 592, 2959–2965 (2014). 24907307 10.1113/jphysiol.2014.272948 PMC4214652

[b68] Neal K. B., Parry L. J. & Bornstein J. C. Strain-specific genetics, anatomy and function of enteric neural serotonergic pathways in inbred mice. J. Physiol. 587, 567–586 (2009). 19064621 10.1113/jphysiol.2008.160416 PMC2670081

[b69] Darcel N. P., Liou A. P., Tome D. & Raybould H. E. Activation of vagal afferents in the rat duodenum by protein digests requires PepT1. J. Nutr. 135, 1491–1495 (2005). 15930458 10.1093/jn/135.6.1491

[b70] Jordi J. *et al.* Specific amino acids inhibit food intake via the area postrema or vagal afferents. J. Physiol. 591, 5611–5621 (2013). 23897232 10.1113/jphysiol.2013.258947 PMC3853499

[b71] Niijima A. Reflex effects of oral, gastrointestinal and hepatoportal glutamate sensors on vagal nerve activity. J. Nutr. 130, 971S–973S (2000). 10736363 10.1093/jn/130.4.971S

[b72] Torii K. & Niijima A. Effect of lysine on afferent activity of the hepatic branch of the vagus nerve in normal and L-lysine-deficient rats. Physiol. Behav. 72, 685–690 (2001). 11337000 10.1016/s0031-9384(01)00426-7

[b73] Muller T. D. *et al.* Ghrelin. Mol. Metab. 4, 437–460 (2015). 26042199 10.1016/j.molmet.2015.03.005 PMC4443295

[b74] Iwakura H. *et al.* Oxytocin and dopamine stimulate ghrelin secretion by the ghrelin-producing cell line, MGN3-1 *in vitro*. Endocrinology. 152, 2619–2625 (2011). 21521750 10.1210/en.2010-1455

[b75] Zhao T. J. *et al.* Ghrelin secretion stimulated by {beta}1-adrenergic receptors in cultured ghrelinoma cells and in fasted mice. Proc. Natl. Acad. Sci. USA 107, 15868–15873 (2010). 20713709 10.1073/pnas.1011116107 PMC2936616

[b76] Broer S. Amino acid transport across mammalian intestinal and renal epithelia. Physiol. Rev. 88, 249–286 (2008). 18195088 10.1152/physrev.00018.2006

[b77] Sigoillot M. *et al.* Optimization of the production of gurmarin, a sweet-taste-suppressing protein, secreted by the methylotrophic yeast Pichia pastoris. Appl. Microbiol. Biotechnol. 96, 1253–1263 (2012). 22307499 10.1007/s00253-012-3897-3

[b78] Nemeth E. F. *et al.* Pharmacodynamics of the type II calcimimetic compound cinacalcet HCl. J. Pharmacol. Exp. Ther. 308, 627–635 (2004). 14593085 10.1124/jpet.103.057273

[b79] Petrel C. *et al.* Modeling and mutagenesis of the binding site of Calhex 231, a novel negative allosteric modulator of the extracellular Ca(2+)-sensing receptor. J. Biol. Chem. 278, 49487–49494 (2003). 14506236 10.1074/jbc.M308010200

[b80] Faure H. *et al.* Molecular determinants of non-competitive antagonist binding to the mouse GPRC6A receptor. Cell calcium. 46, 323–332 (2009). 19836834 10.1016/j.ceca.2009.09.004

[b81] Bleasdale J. E. *et al.* Selective inhibition of receptor-coupled phospholipase C-dependent processes in human platelets and polymorphonuclear neutrophils. J. Pharmacol. Exp. Ther. 255, 756–768 (1990). 2147038

[b82] Davidson G. A. & Varhol R. J. Kinetics of thapsigargin-Ca(2+)-ATPase (sarcoplasmic reticulum) interaction reveals a two-step binding mechanism and picomolar inhibition. J. Biol. Chem. 270, 11731–11734 (1995). 7744817 10.1074/jbc.270.20.11731

[b83] Young S. H., Rey O., Sternini C. & Rozengurt E. Amino acid sensing by enteroendocrine STC-1 cells: role of the Na+-coupled neutral amino acid transporter 2. Am. J. Physiol. Cell Physiol. 298, C1401–1413 (2010). 20219951 10.1152/ajpcell.00518.2009 PMC2889636

[b84] Granata R. *et al.* Obestatin promotes survival of pancreatic beta-cells and human islets and induces expression of genes involved in the regulation of beta-cell mass and function. Diabetes. 57, 967–979 (2008). 18162507 10.2337/db07-1104

[b85] Thorens B. *et al.* Cloning and functional expression of the human islet GLP-1 receptor. Demonstration that exendin-4 is an agonist and exendin-(9-39) an antagonist of the receptor. Diabetes. 42, 1678–1682 (1993). 8405712 10.2337/diab.42.11.1678

[b86] Carrillo J., Agra N., Fernandez N., Pestana A. & Alonso J. Devazepide, a nonpeptide antagonist of CCK receptors, induces apoptosis and inhibits Ewing tumor growth. Anti-cancer drugs. 20, 527–533 (2009). 19407653 10.1097/CAD.0b013e32832c3a4f

[b87] Chang R. S. & Lotti V. J. Biochemical and pharmacological characterization of an extremely potent and selective nonpeptide cholecystokinin antagonist. Proc. Natl. Acad. Sci. USA 83, 4923–4926 (1986). 3014520 10.1073/pnas.83.13.4923 PMC323856

[b88] Tulipano G. *et al.* Characterization of new selective somatostatin receptor subtype-2 (sst2) antagonists, BIM-23627 and BIM-23454. Effects of BIM-23627 on GH release in anesthetized male rats after short-term high-dose dexamethasone treatment. Endocrinology. 143, 1218–1224 (2002). 11897676 10.1210/endo.143.4.8716

[b89] Verhulst P. J. *et al.* Role of ghrelin in the relationship between hyperphagia and accelerated gastric emptying in diabetic mice. Gastroenterology. 135, 1267–1276 (2008). 18657539 10.1053/j.gastro.2008.06.044

