# Supplementary material for: Chemosensory signalling pathways involved in sensing of amino acids by the ghrelin cell
Source: Sci Rep. 2015 Oct 29;5:15725. doi: 10.1038/srep15725 (PMC4625164; doi:10.1038/srep15725)
Supplement: Supplementary Information [file srep15725-s1.pdf]

Chemosensory signalling pathways involved in sensing of amino acids by the ghrelin cell

L. Vancleef[1], T. Van Den Broeck[1], T. Thijs[1], S. Steensels[1], L. Briand[2], J. Tack[1], I. Depoortere[1,*]

[1] Gut Peptide Research Lab, Translational Research Center for Gastrointestinal Disorders, Department of Clinical & Experimental Medicine, University of Leuven, Leuven, 3000, Belgium

[2] INRA UMR1324, CNRS UMR6265, Université de Bourgogne, Centre des Sciences du Goût et de l'Alimentation, F-21000, Dijon, France

*inge.depoortere@med.kuleuven.be

Address for Correspondence:

Prof I. Depoortere, PhD

Translational Research Center for Gastrointestinal Disorders

Gut Peptide Research Lab

Gasthuisberg O&N1, box 701

3000 Leuven

Belgium

E-mail: inge.depoortere@med.kuleuven.be

Tel: +32-16-330675

Fax: +32-16-330723

Supplementary figure 1

Figure S1

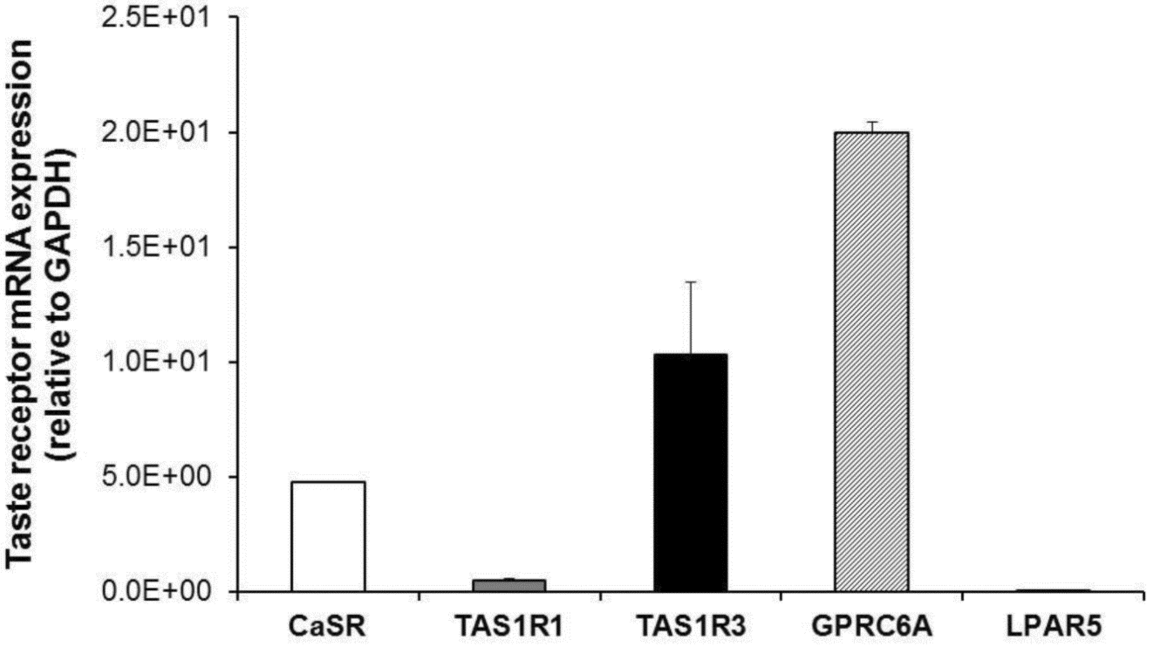

Supplementary figure S1. Expression of amino acid/peptide taste receptors in the ghrelinoma cell line. Relative mRNA expression of the 3 amino acid taste receptors (CaSR, TAS1R1-TAS1R3 and GPRC6A) and a peptide receptor (LPAR5) in the ghrelinoma cell line, MGN3-1 (n=2/receptor) as determined by real time RT-PCR.

Supplementary figure 2

Figure S2

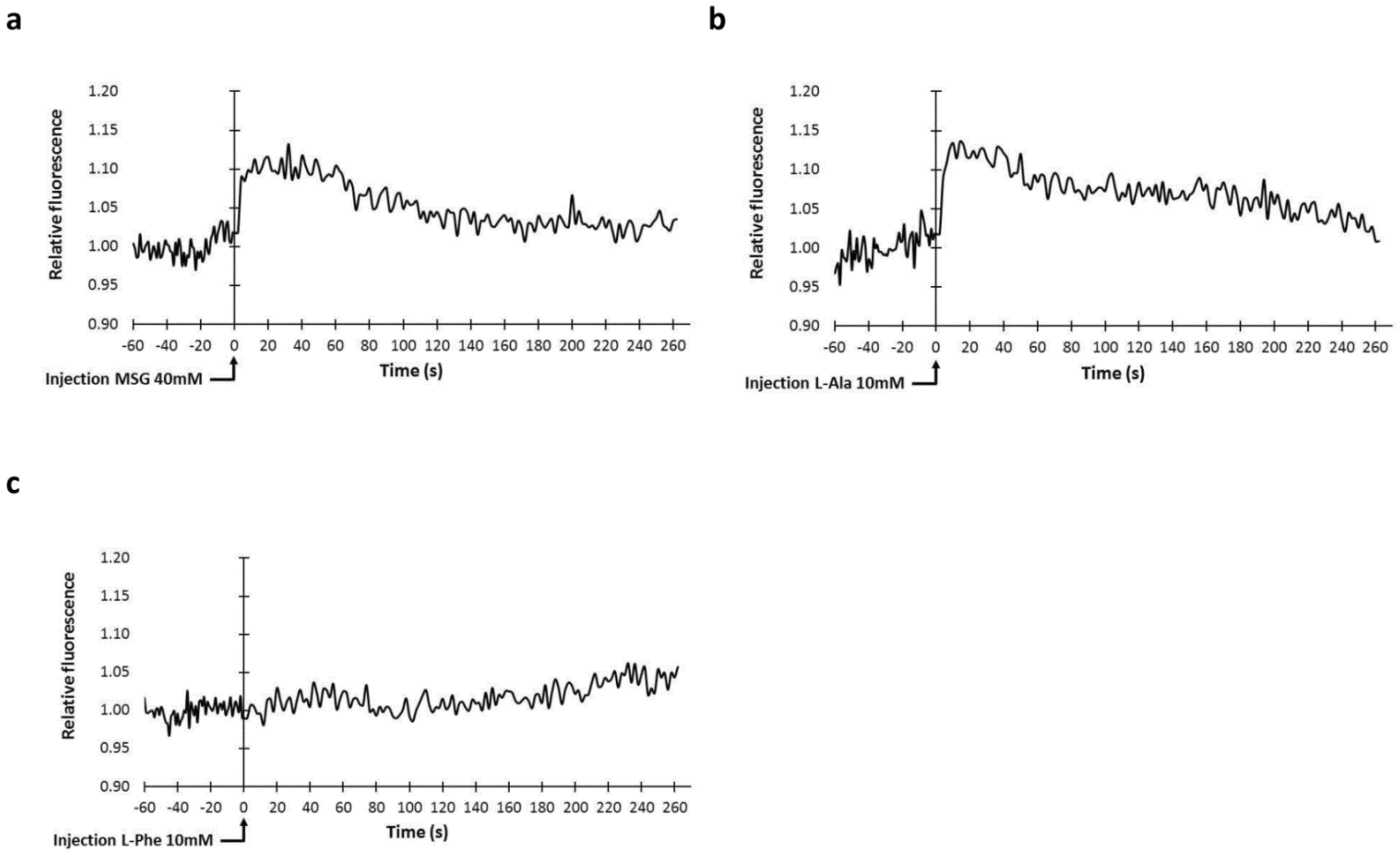

Supplementary figure S2. Intracellular Ca^{2+} responses induced by amino acids in MGN3-1 cells. Representative tracing of the rise in intracellular Ca^{2+} after administration of (**A**) MSG 40mM, (**B**) L-Ala 10mM and (**C**) L-Phe 10mM to MGN3-1 cells.
